# Herpes Zoster and Paralysis

**Published:** 1907-10-26

**Authors:** 


### Illustrative Cases.

### HERPES ZOSTER AND PARALYSIS.

Herpes zoster is generally regarded as a tropho-neurosis, secondary to an inflammatory lesion in the corresponding posterior nerve-root ganglia. There is still discussion as to whether the trophic changes are the direct result of the nerve lesion, or whether they are indirect, viâ the blood-vessels and the vaso-motor nerves. There is still discussion also as to whether the eruption follows nerve trunks and branches, or whether the distribution is by spinal cord segments independently of the actual nerve fibres in the skin. The majority of cases seem to have the eruption in fairly close relationship to nerve branches rather than cord segments, and in most cases the fact that the sensory nerves are involved is very much impressed both on the patient and on the doctor by the intense pain which accompanies the eruption; this pain is not in the herpetic patches in the skin, but in the nerves beneath the skin, often affecting areas not covered by any part of the eruption. The pains are often referred to the areas corresponding to spinal-cord segments, but the eruption is rather restricted to the areas supplied by the peripheral nerves whose posterior root ganglia are affected.

### Muscular Paresis.

Little has been written of any affection of the anterior nerve roots, or motor nerve trunks simultaneously with the trouble in the posterior nerve roots. Probably, therefore, it is not common for pareses of muscles to be associated with herpes zoster. Nevertheless, it is interesting to note that motor trouble can and does occur in some cases. When one sees one's first case of the kind it is rather alarming, because the paresis may suggest that there is far more than mere herpetic inflammation the matter, and that the original diagnosis is insufficient. This is particularly the case in cervical or trigeminal herpes, when facial paralysis or ophthalmoplegia may accompany or follow the eruption, and suggest cerebral tumour, gumma, or other gross lesion within the cranium. The prognosis of the paresis occasionally associated with herpes is very fair, however, the rate of recovery varying inversely with the degree to which the nerve fibres have degenerated; in mild cases the paresis may be transient; in severer cases, recovery begins some 12 or 14 weeks after the paralysis sets it—that is to say, in about the same time as physiologists find it takes divided nerve fibres to regenerate from their central ends.

### Three Illustrative Cases.

The following are three cases of local paralysis associated with herpes zoster. The first is an example of partial ophthalmoplegia accompanying supra-orbital herpes; the other two exemplify facial paralysis associated with cervical zona.

Henry B., aged 62, had been quite well hitherto, when he began to complain of irritation in his left eye and severe pains over the left orbit, and extending up to the parietal region on the left side of his head. All that was noticed at this time was a condition that looked like ordinary acute conjunctivitis in the left eye. This was treated with boric acid lotion, the eye being kept from exposure to light by a loose fitting blinker. Next day the whole of the skin above the left eye had become bright pinkish red, and slightly puffy, and now for the first time herpes was suspected. Two days later there was a very extensive and severe herpetic eruption all over the left side of the forehead, and extending back as far as the remotest branches of the supra-orbital nerve reach. The pain was intense, rendering continued sleep an impossibility for many nights in succession. No actual vesicles occurred in the conjunctiva itself, and no branches of any other divisions of the fifth cranial nerve were involved. Simple treatment by boric acid fomentations relieved the pain more than anything else; the fomentations were continued for about a week, when they were changed for a dry boric acid powder application. About the tenth day, when the herpes was subsiding, the patient noticed that he could not get his left eyelid up to its full extent. At first this was attributed to the photophobia from which he had been suffering; but day by day the weakness of the eyelid increased instead of diminishing, until during the third week from the beginning of the mischief he could not raise his left eyelid voluntarily at all. He could push it up with his finger, and could then see perfectly well. There was no paresis of any of the muscles moving the eyeball, nor any of the intraocular muscles. The ptosis was a great trouble to him, and at first a lesion at the base of the brain was suspected—a small local hæmorrhage, for example. The trouble slowly but surely subsided, however, and in about three months' time the ptosis had completely disappeared, and the patient seemed absolutely well again in all respects. There was no evidence of granular kidney nor of other cause of cerebral hæmorrhage. There were no general symptoms of cerebral tumour; the diagnosis seemed to be ordinary supra-orbital zona complicated by paresis of the levator palpebræ superioris.

### The Second Case.

David K., aged 52, an ordinary mechanic, who had been by no means teetotal, but who had otherwise been in excellent general health, began to complain of an intense pain in the back of his neck, chiefly on the left side. He had received no injury there, and was quite unable to account for its origin. The least movement or pressure caused such agony that he could not go on with his work. At that time there was nothing abnormal to be seen, but next day the skin over the whole of the painful region was tense, blushed, and shiny. A hand held over it seemed to burn; there was also some fever, and the appetite was lost. Two days later typical zona appeared, the groups of vesicles covering the left cervico-occipital region. The auricular, mastoid, and transverse cervical branches of the cervical plexus were well picked out, and there were a few vesicles over the descending branches, except the supra-clavicular and supraacromial. The number of vesicles was far greater in the upper than in the lower part of the neck. Eight days later it was first noticed that the left side of the patient's face looked queer, and a day later there was typical and complete infranuclear paralysis of the left facial nerve, except for preservation of taste. There was no affection of any other cranial nerve; even the left auditory nerve was quite natural. There was no otitis media, nor could the facial paralysis be attributed to anything but the attack of zona. The latter dried up in a week or two, but the paralysis of the face persisted much longer; it was still discernible, though very much improved, seven weeks later. Ultimately it got perfectly well.

### The Third Case.

Charles K., aged 72, was a very similar case. He had never ailed until he developed acute pain, accompanied by spots, in the left cervico-occipital region. When seen there was extensive pyodermia of the whole of the herpetic area, but it was clear that the condition was one of zona, affecting the upper branches of the left superficial cervical plexus. There was intense irritation, but at the same time deficient cutaneous sensibility in the region involved, and the hair fell out over the whole of it, but not elsewhere. When he had been a few days under observation, it was noticed that he could not close his left eye properly, and next day he had complete paralysis of the left facial nerve, with loss of taste in the left side of his tongue. No other cranial nerve was affected; there had been no traumatism, syphilis, lead poisoning, exposure to draught, otitis media, or other obvious cause for the facial paralysis. It seemed to be directly due to the herpes zoster in the neck.

### Conclusion.

It seems not unlikely that the acute inflammatory exudation within as well as upon the skin in the last two cases affected much deeper structures also, and amongst them the periosteum at the stylo-mastoid foramen; this might be the explanation of the facial paralysis in the case where there was no loss of taste. To account for the loss of taste in the third case one might assume that the inflammation extended a good deal further into the aqueductus Fallopii, reaching as far as the chorda tympani; in the first case perhaps the inflammatory exudate actually infiltrated the nerve going to the levator palpebræ superioris. However it arises, it is quite worthy of being noted that herpes zoster of the supra-orbital nerve may be directly associated with troublesome ptosis, and that the same affection in the superior cervical region may be the cause of infranuclear facial paralysis.

